# CCNB2 as a potential biomarker of bladder cancer via the high throughput technology

**DOI:** 10.1097/MD.0000000000032825

**Published:** 2023-02-10

**Authors:** Lei Zhang, Bin Liu, Jianzhi Su

**Affiliations:** a Department of Urology Surgery, Fuxing Hospital Affiliated to Capital Medical University, Xicheng District, Beijing, China; b Department of Urology Surgery, The Fourth Hospital of Hebei Medical University, Hebei, PR China.

**Keywords:** bioinformatics technology, bladder cancer, CCNB2, gene targets

## Abstract

Bladder cancer and oral squamous cell carcinoma (OSCC) seriously affect people’s health. However, the relationship between bladder cancer and OSCC remains unclear. Got GSE138206, GSE146483, GSE184616, and bladder cancer datasets GSE65635, GSE100926 from Gene Expression Omnibus database. Weighted gene co-expression network analysis was used to identify the significant module. Functional enrichment analysis was performed via the Gene Ontology analysis and Kyoto Encyclopedia of Genes and Genomes. Furthermore, the Gene Set Enrichment Analysis was also used to complete the enrichment analysis. Comparative Toxicogenomics Database found most relevant diseases to core genes. TargetScan is used to forecast analysis of microRNA and target genes. In Gene Ontology analysis, differentially expressed genes were mostly concentrated in cell differentiation, extrallular region, structural molecule activity, and actin binding. In Kyoto Encyclopedia of Genes and Genomes analysis, the differentially expressed genes were mainly enriched in PI3K-Akt signaling pathway, pathway in cancer, and extracellular matrix-receptor interaction. Seven hub genes (cyclin B2 [CCNB2], TK1, CDC20, PCNA, CKS1B, CDCA5, MCM4) were obtained. Hub genes (CCNB2, CDC20) are highly expressed in OSCC and bladder cancer samples. CCNB2 was one common oncogene of bladder cancer and OSCC.

## 1. Introduction

Bladder cancer is the tenth most common cancer in the world.^[1]^ Bacillus Calmette-Guerin vaccine is an effective drug for the treatment of high-risk non-muscular invasive bladder cancer and carcinoma in situ, but the overall adverse reaction rate is as high as 71.8%.^[2]^ Meanwhile, oral cancer is a very common malignant tumor with high recurrence rate and high drug resistance.^[3]^ Oral cancer is diagnosed in 600,000 new cases worldwide each year, with a mortality rate of 40 to 50 percent.^[4]^ Oral cancer is prone to distant metastasis. Patients have a poor prognosis and a low quality of life.^[5–7]^ The relationship between oral cancer and bladder cancer is still unclear, so it is important to further study the molecular mechanism of oral cancer and bladder cancer.

Bioinformatics is an effective combination of biology and computer, mainly through the comprehensive application of mathematics and information science, and other fields to explore the molecular targets of biological diseases.^[8]^ High-throughput sequencing is becoming widely used to find candidate genes for diseases.^[9]^ High-throughput sequencing techniques sequence hundreds of thousands to millions of DNA molecules at a time, and high-throughput sequencing makes it possible to analyze the transcriptome and genome of a species in detail.^[10,11]^ Cyclin B2 (CCNB2) is closely associated with tumors. CCNB2, a member of the cyclin family, plays a major role in G2/M transformation and is upregulated in many cancers.^[12]^ CCNB2 is highly expressed in glioma and is associated with poor prognosis, while decreased CCNB2 expression inhibits the invasion and metastasis of cancer.^[13]^ However, the relationship between CCNB2 and oral and bladder cancers remains unclear.

Therefore, this paper intends to use bioinformatics technology to screen the hub genes between oral cancer, bladder cancer, and normal tissues, and conduct enrichment analysis pathway analysis using public data sets to verify the significant role of CCNB2 in oral cancer and bladder cancer.

## 2. Methods

### 2.1. Oral squamous cell carcinoma (OSCC) dataset

Got OSCC datasets GSE138206, GSE146483, GSE184616 and bladder cancer dataset GSE65635, and GSE100926 from Gene Expression Omnibus database (http://www.ncbi.nlm.nih.gov/geo/) generated by GPL570, GPL17077, GPL24676. GSE138206 included 6 OSCC and 6 normal tissue samples; GSE146483 included 8 OSCC and 3 normal tissue samples. GSE184616 included 15 OSCC and 15 normal tissue samples. GSE65635 includes 8 bladder cancer and 4 normal tissue samples, and GSE100926 includes 3 bladder cancer and 3 normal tissue samples. Differentially expressed genes (DEGs) to identify OSCC and bladder cancer.

### 2.2. To batch processing

OSCC datasets GSE138206, GSE146483, and GSE184616 were combined using R package inSilicoMerging (Ross Ihaka and Robert Gentleman, University of Auckland, New Zealand) [DOD:10.1186/1471-2105-13-335] to obtain merge matrix, bladder cancer dataset GSE65635, GSE100926 were also combined using R package inSilicoMerging. Furthermore, we used the remove BatchEffect function of the R package limma (version 3.42.2) (Ross Ihaka and Robert Gentleman) to remove the batch effect, and finally obtained matrix after removing the batch effect, which was applied to subsequent analysis.

### 2.3. Screening of DEGs

R package “limma” was used for probe summary and background correction of merging matrices for GSE138206, GSE146483, and GSE184616. Used Benjamini–Hochberg method to set raw *P* values. Used fold change to get a false discovery rate. The cutoff criterion for DEG was *P* < .05. Volcano plot was made to obtain DEGs.

### 2.4. Weighted gene co-expression network analysis (WGCNA)

Top 50% of genes with smallest median absolute deviation were acquired and excluded. For all genes in pairs perform Pearson correlation matrix and average chain method, using power function a|mn=| C|mn |^β^ to build weighted adjacency matrix. After choosing the soft threshold parameter, converted the adjacency matrix to the topological overlap matrix. Average linkage hierarchical clustering was performed, minimum size (genome) was 30. Sensitivity was set to 3. We calculated the phase divergence of module feature genes, incorporating modules with distances <0.25. At the same time, we also predicted the inter-relationship of genes in the module to obtain core genes.

### 2.5. Protein-protein interaction (PPI) network

List of genes was input into Search Tool for the Retrieval of Interacting Genes (https://cn.string-db.org/cgi/input.pl) database to build a PPI network (confidence > 0.4) for predicting core genes. PPI network was imported into Cytoscape software (U.S. National Institute of General Medical Sciences). Used Maximal Clique Centrality algorithm to calculate 10 best correlation genes and take intersection, core gene list was exported after visualization.

### 2.6. Functional enrichment analysis

This study will Wayne figure out the difference of gene list input Kyoto Encyclopedia of Genes and Genomes (KEGG) rest (https://www.kegg.jp/kegg/rest/keggapi.html), get latest KEGG pathway gene annotation; used R package clusterProfiler (version 3.14.3) (Ross Ihaka and Robert Gentleman) for enrichment analysis to get results of gene set enrichment. Gene Ontology (GO) annotation was performed by R package “org.Hs.e.g.db (version 3.1.0)” (Ross Ihaka and Robert Gentleman). And the minimum gene set was 5, and maximum gene set was 5000. *P* value <.05, and false discovery rate <0.25 are defined as statistical significance.

In addition, we use Metascape database (http://metascape.org/gp/index.html), for above differences in gene enrichment of function analysis and export list.

### 2.7. Gene Set Enrichment Analysis (GSEA) analysis

We got GSEA software (version 3.0) from GSEA (DOI: 10.1073/ pnas. 0506580102, http://software.broadinstitute.org/gsea/index.jsp) website. Divided samples into 2 groups based on OSCC and normal tissues, derived from Molecular Signatures Database (DOI: 10.1093/ bioinformatics/btr260, http://www.gsea-msigdb.org/gsea/downloads.jsp) to download c2.cp.kegg.v7.4.symbols.gmt. Based on gene expression profile and phenotype grouping, minimum gene set 5, maximum gene set 5000, 1000 resampling times.

### 2.8. Heat map of gene expression

By R package heatmap (Ross Ihaka and Robert Gentleman) to make a heatmap of expression degree of hub genes found by common DEGs of OSCC debatching merger matrix and Benson Latin American Collection debatching merger matrix.

### 2.9. Comparative Toxicogenomics Database (CTD) analysis

We entered core genes into CTD website, found most relevant diseases to core genes, used Excel (Microsoft, Redmond, WA) to draw differential expression radar map of each gene.

### 2.10. miRNA

TargetScan (www.targetscan.org) is used to forecast analysis of microRNA and target genes. Enter core gene into website, search it, and get miRNA regulating central DEGs.

## 3. Results

### 3.1. Differential expression gene analysis

**Figure 1.**
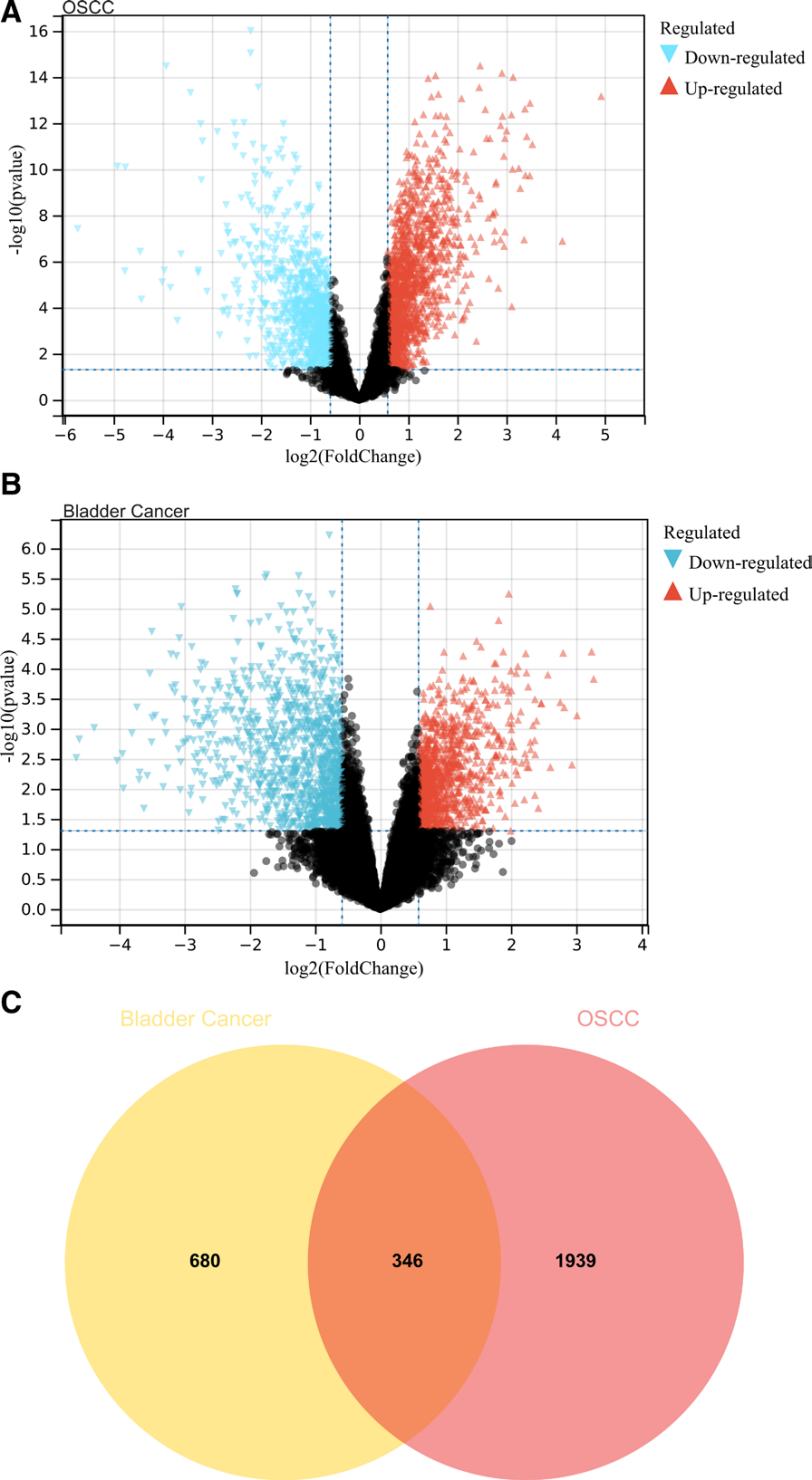
Functional enrichment analysis of DEGs. (A) 2285 DEGs were identified. (B) 1026 DEGs were identified. (C) Venn diagram was drawn for the 2 DEGs groups. DEGs = differentially expressed genes.

Two thousand two hundred eighty-five DEGs were found based on DEGs identified in debatching merger matrix of GSE138206, GSE146483, GSE184616 (Fig. 1A),1026 DEGs were found based on DEGs identified in debatching merger matrix of GSE65635, GSE100926 (Fig. 1B). And there are 346 intersections of 2 groups of DEGs (Fig. 1C).

### 3.2. Functional enrichment analysis

#### 3.2.1. Functional enrichment analysis of DEGs.

**Figure 2.**
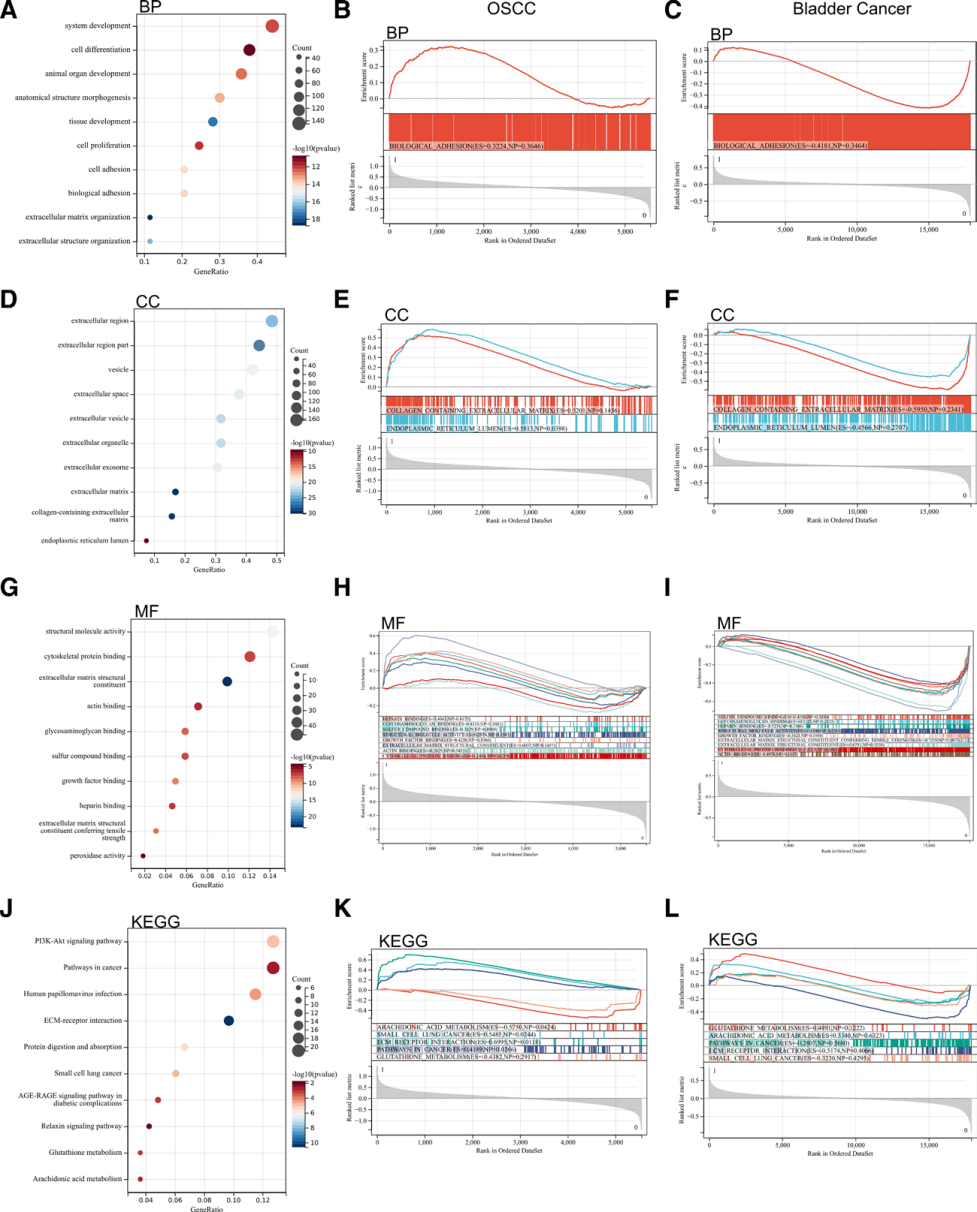
GO and KEGG functional enrichment analysis results. GO = Gene Ontology, KEGG = Kyoto Encyclopedia of Genes and Genomes.

In GO analysis, they were mostly concentrated on the organization of system development, cell differentiation, extracellular region, structural molecule activity, actin binding. In KEGG analysis, the DEGs were mainly enriched in the PI3K-Akt signaling pathway and pathway in cancer. (Fig. 2).

We performed GSEA enrichment analysis on whole genome to find possible enrichment terms in non-DEGs. The results are shown in the figure, and the enrichment terms are similar to GO and KEGG enrichment terms for DEGs (Fig. 2).

#### 3.2.2. Enrichment analysis by Metascape.

**Figure 3.**
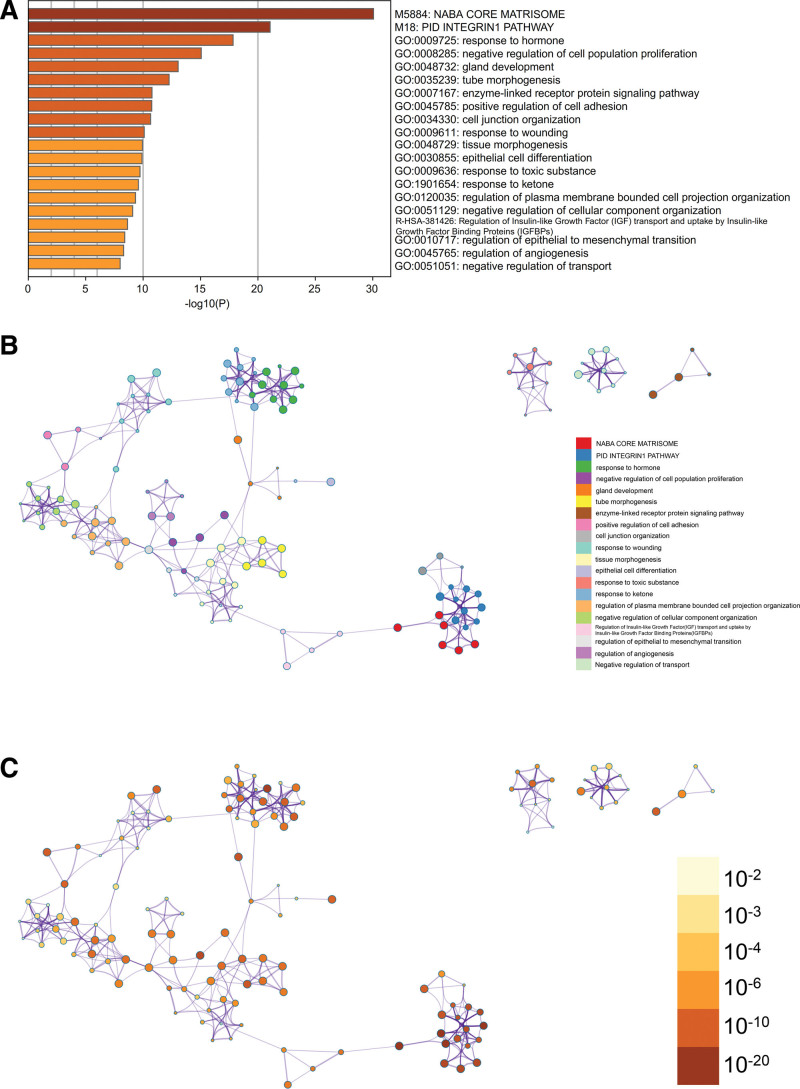
Enrichment analysis by Metascape. (A) GO enrichment term. (B) Enriched networks colored by enriched terms. (C) Enriched networks colored by *P* values. GO = Gene Ontology.

**Figure 4.**
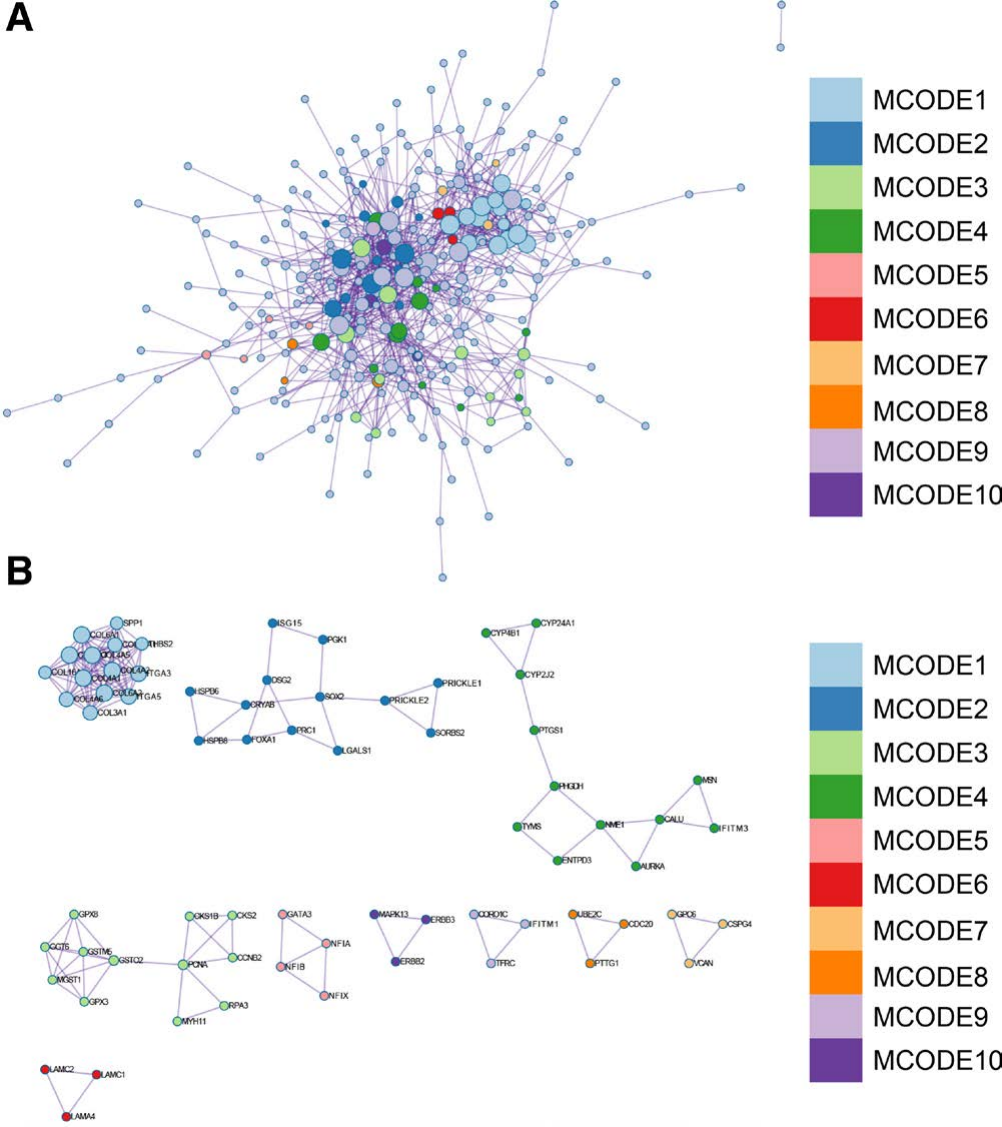
(A) Enrichment analysis by Metascape. (B) Protein-protein interaction network and MCODE components identified in the gene lists.

The content enriched by Metascape includes GO-enriched terms (Fig. 3A) and has an enriched network colored by enriched terms and *P* value (Fig. 3B and C and Fig. 4).

### 3.3. WGCNA analysis

**Figure 5.**
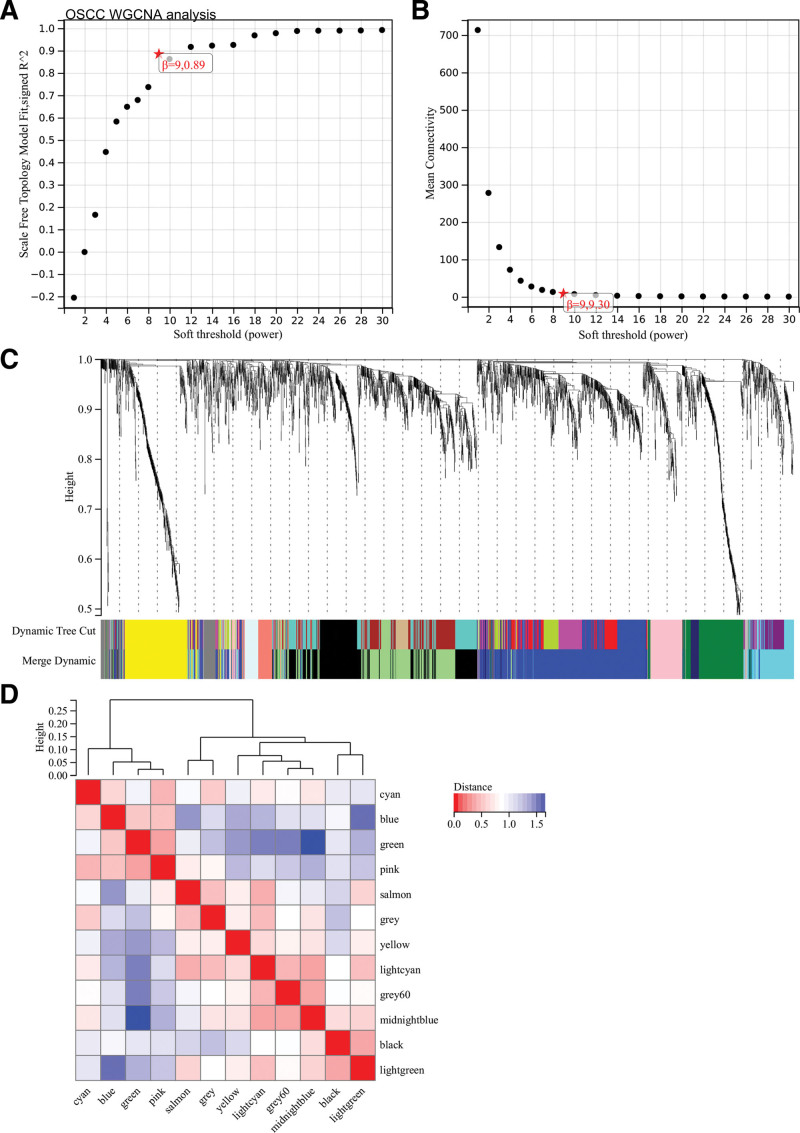
WGCNA analysis of OSCC. (A) *β* = 9,0.89. (B) *β* = 9,9.30. (C) Hierarchical clustering trees of all genes were constructed and 12 important modules were generated. (D) Interaction between modules. OSCC = oral squamous cell carcinoma, WGCNA = weighted gene co-expression network analysis.

**Figure 6.**
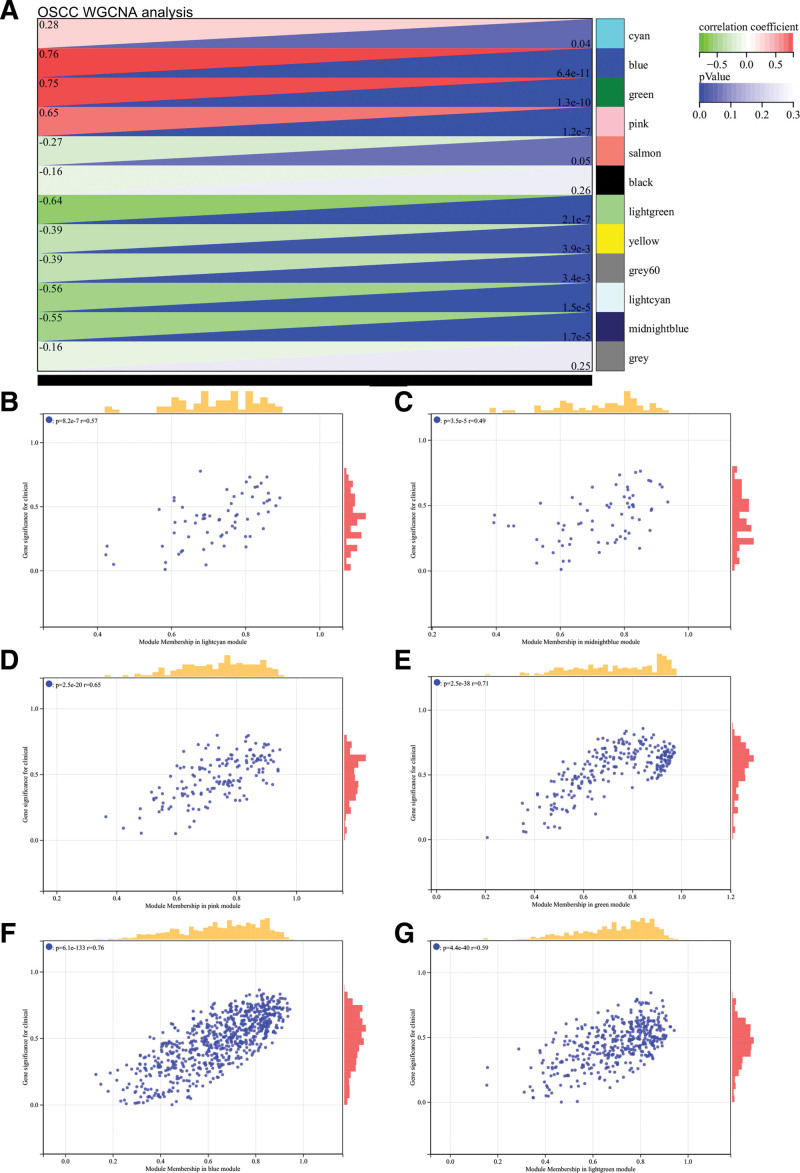
WGCNA analysis of OSCC. (A) Heat map of module and phenotype correlation. (B–G) Scatter plot of GS and MM correlation of related hub genes. GS = gene significance, MM = module membership, OSCC = oral squamous cell carcinoma, WGCNA = weighted gene co-expression network analysis.

Through the WGCNA of the OSCC, set soft threshold power at 9, which is lowest power for scale-free topological fit index of 0.9 (Fig. 5A and B). Hierarchical clustering trees were constructed for all genes and yielded 22 significant modules (Fig. 5C). Analyzed interaction between modules (Fig. 5D). Heatmaps of module and phenotype correlations (Fig. 6A) and scatter plots of gene significance and module membership (MM) correlations of related hub genes (Figs. 6B–G) were also generated.

We calculated module characteristic vector correlation with the expression of genes for MM, according to cutting standard (|MM| > 0.8), confirmed 12 high connectivity genes in clinically significant modules as pivot genes.

**Figure 7.**
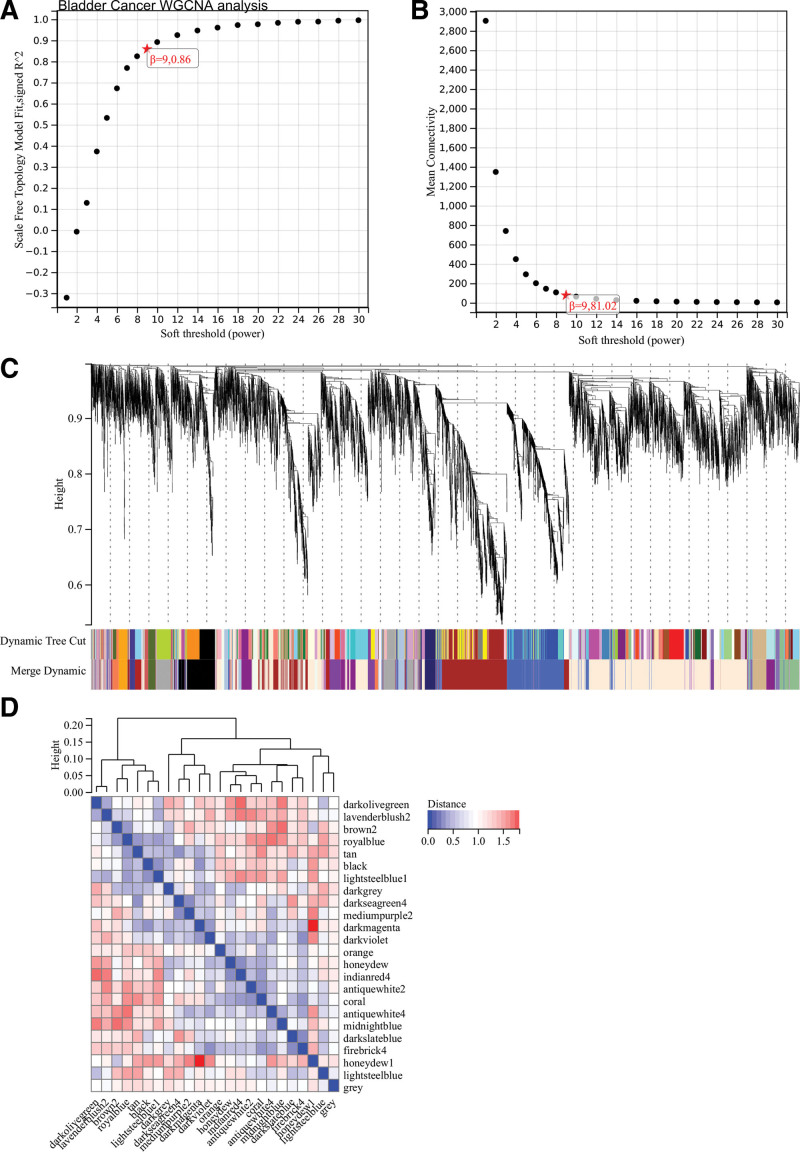
WGCNA analysis of BLCA. (A) *β* = 9,0.86. (B) *β* = 9,81.2. (C) Hierarchical clustering trees of all genes were constructed and 24 important modules were generated. (D) Interaction between modules. BLCA = bladder cancer, WGCNA = weighted gene co-expression network analysis.

**Figure 8.**
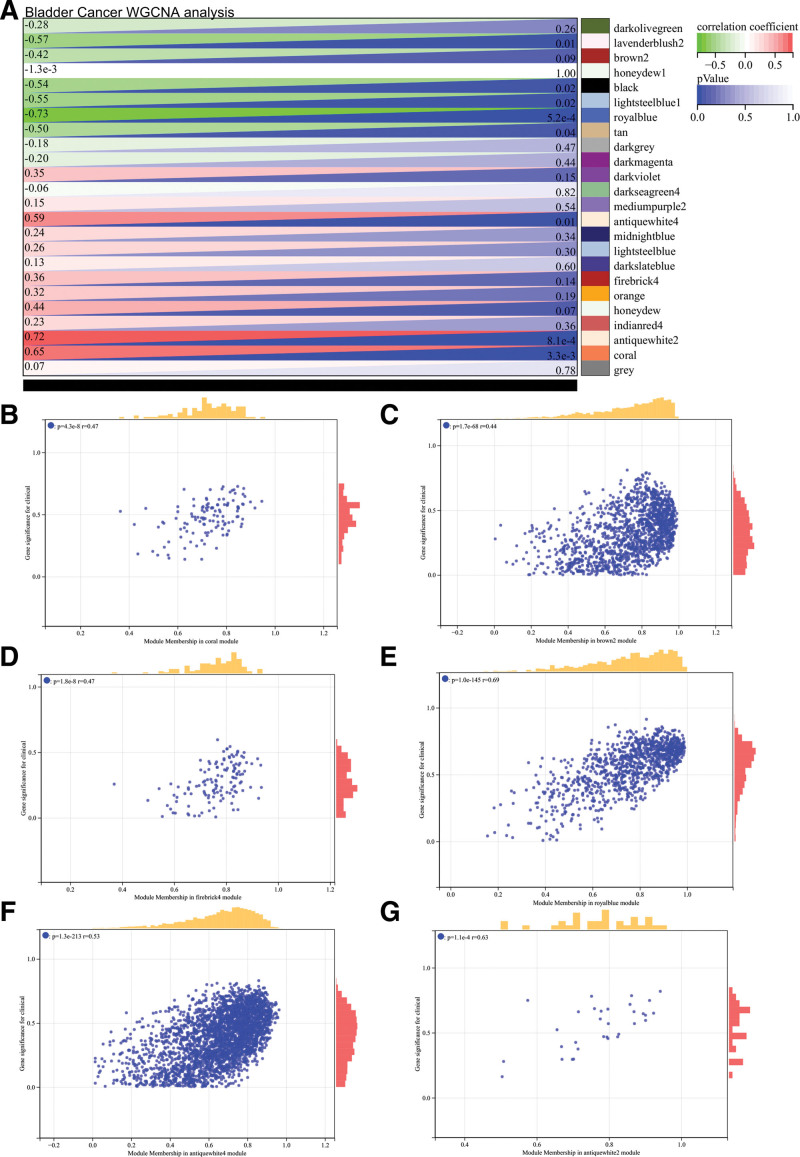
WGCNA analysis of BLCA. (A) Heat map of module and phenotype correlation. (B–G) Scatter plot of GS and MM correlation of related hub genes. BLCA = , GS = gene significance, MM = module membership, WGCNA = weighted gene co-expression network analysis.

Furthermore, in the WGCNA analysis of BLCA, hierarchical clustering trees of all genes were constructed and 24 important modules were generated (Fig. 7). Heat map of module and phenotype correlation was analyzed, and scatter plot of gene significance and MM correlation of related hub genes were generated (Fig. 8).

### 3.4. PPI network

**Figure 9.**
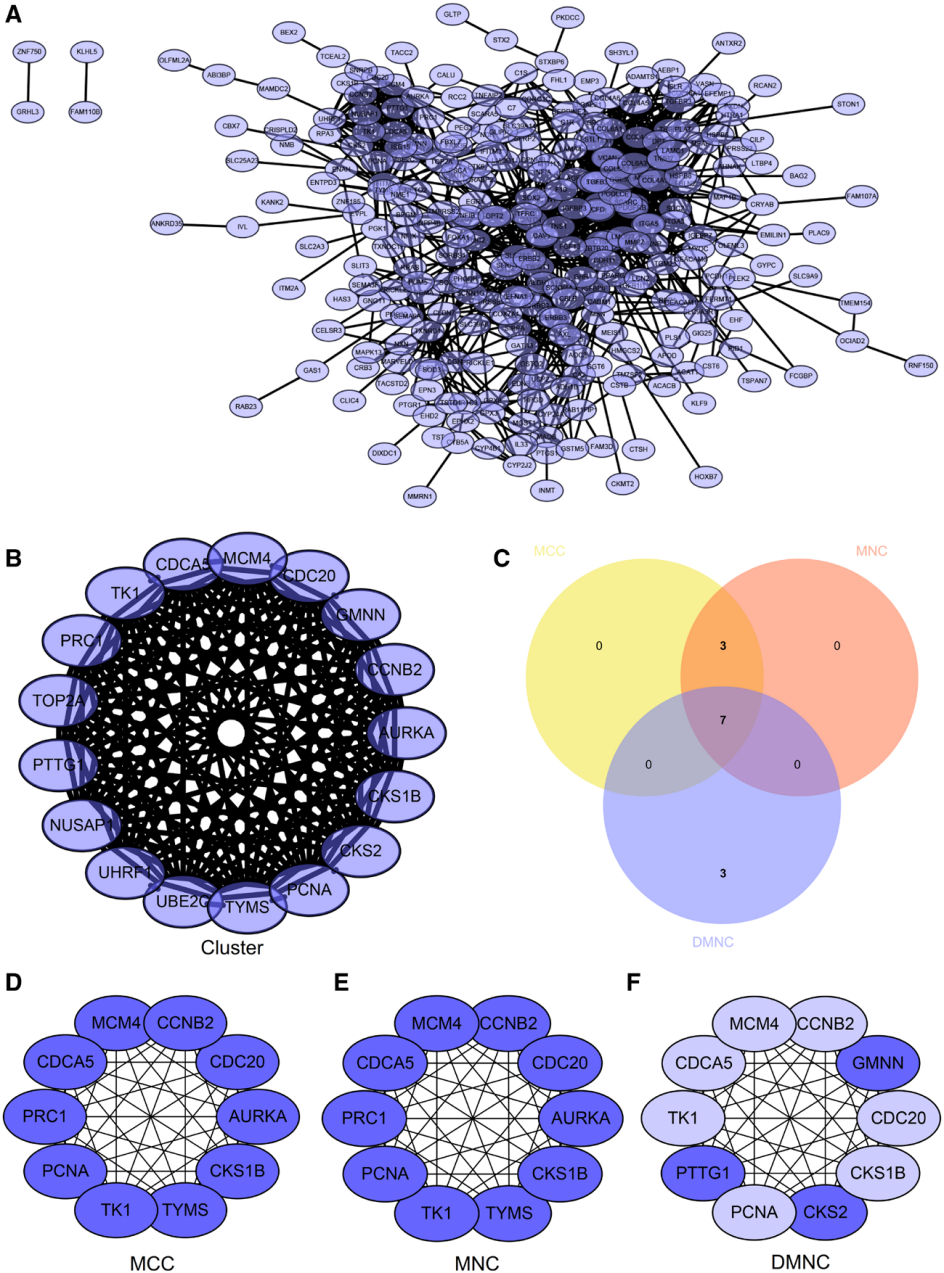
Construction and analysis of protein-protein interaction (PPI) network. (A) PPI network of DEGs. (B) Core gene clusters: CLUSTER. (C) Venn diagram and took the intersection to obtain 7 core genes (CCNB2, TK1, CDC20, PCNA, CKS1B, CDCA5, MCM4). (D) The algorithm of MCC identified the central genes. (E) The MNC algorithm identified the central genes. CCNB2 = cyclin B2, DEGs = differentially expressed genes, MCC = Maximal Clique Centrality, MNC = Maximum Neighborhood Component.

PPI network was presented (Fig. 9A), and 1 core module using MCODE algorithm (Fig. 9B) was obtained. Venn diagram was drawn and intersection was taken (Fig. 9C). Seven hub genes (CCNB2, TK1, CDC20, PCNA, CKS1B, CDCA5, MCM4) were obtained. Used Maximal Clique Centrality, MNC, DMNC algorithms to confirm hub genes (Fig. 9D–F).

### 3.5. Heat map of gene expression

**Figure 10.**
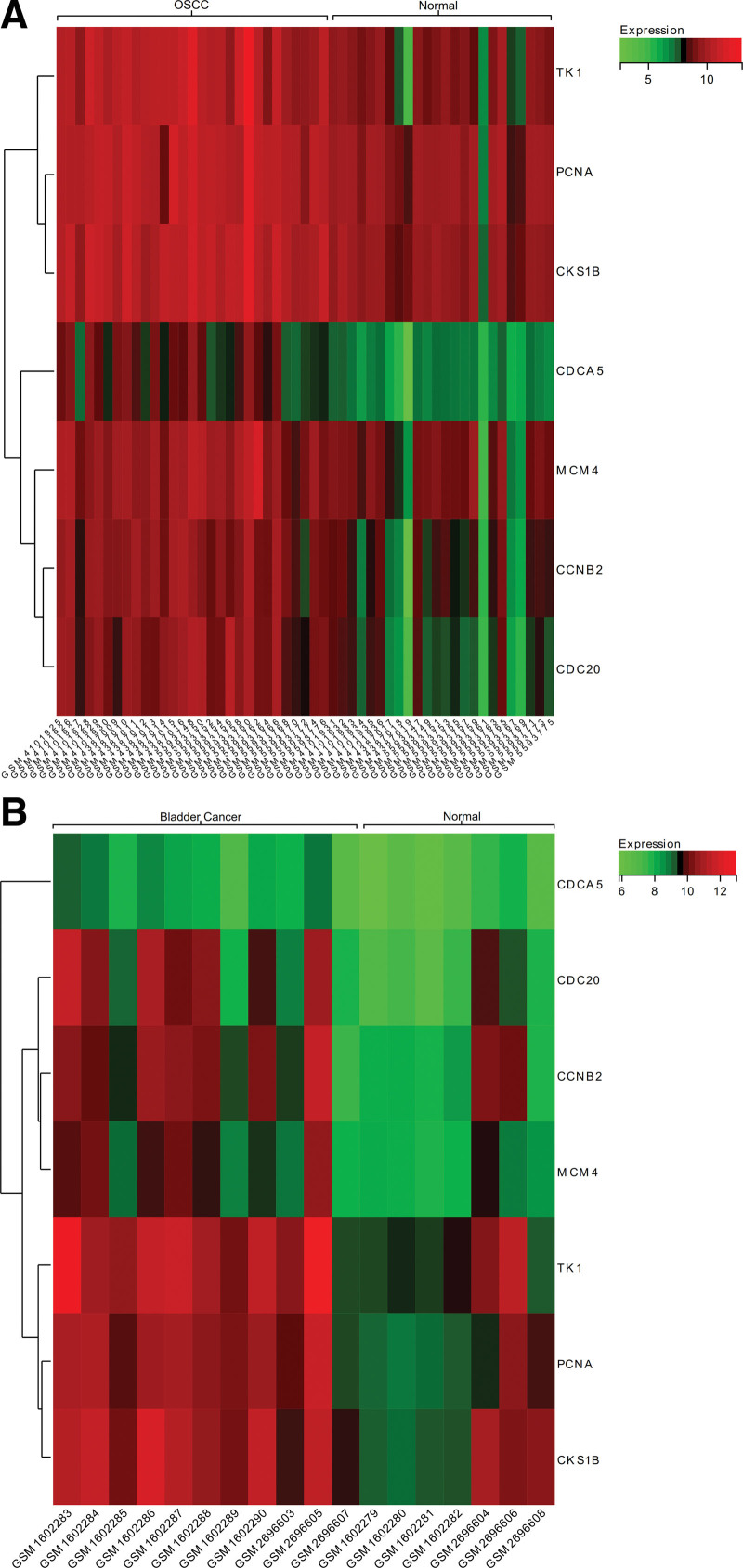
Heat map of OSCC and BLAC gene expression. (A) Core genes were differentially expressed between OSCC and normal tissue samples. (B) Core genes were differentially expressed between BLAC and normal tissue samples. BLAC = Benson Latin American Collection, OSCC = oral squamous cell carcinoma.

Difference in expression of hub genes between OSCC and normal tissues is shown in heat map, they were also shown between Benson Latin American Collection and normal tissues. Hub genes (CCNB2, CDC20) are highly expressed in OSCC and bladder cancer samples (Fig. 10).

### 3.6. Expression analysis

**Figure 11.**
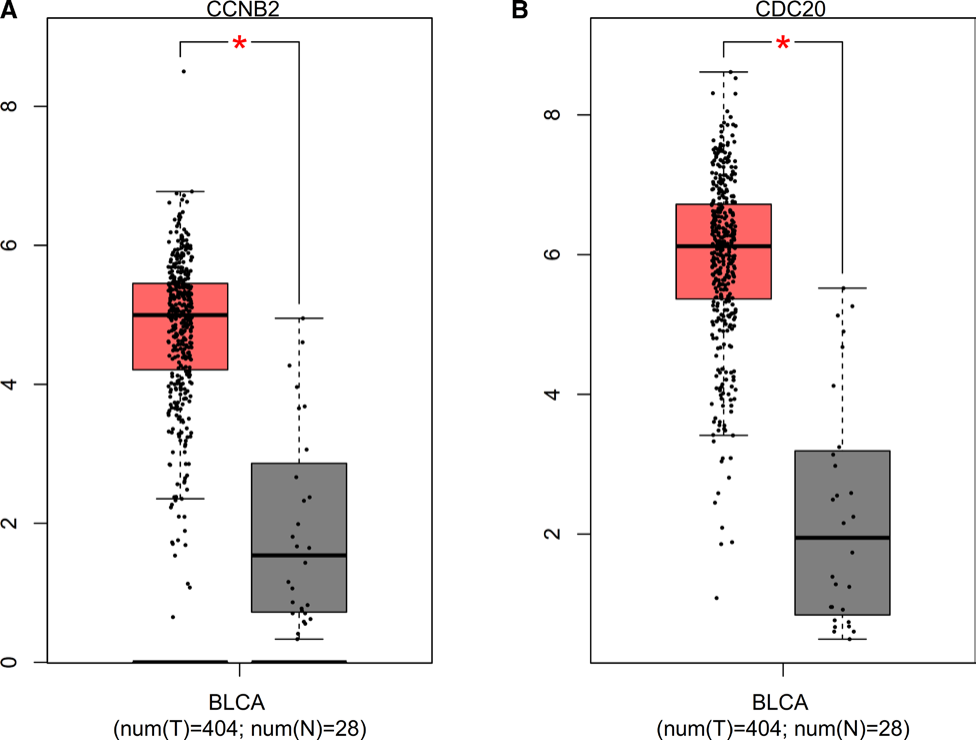
Expression of hub genes. (A) CCNB2. (B) CDC20.*P < .05. CCNB2 = cyclin B2.

Expression of CCNB2 and CDC20 were upregulated in the BLCA compared with the normal tissues (Fig. 11).

### 3.7. CTD analysis

**Figure 12.**
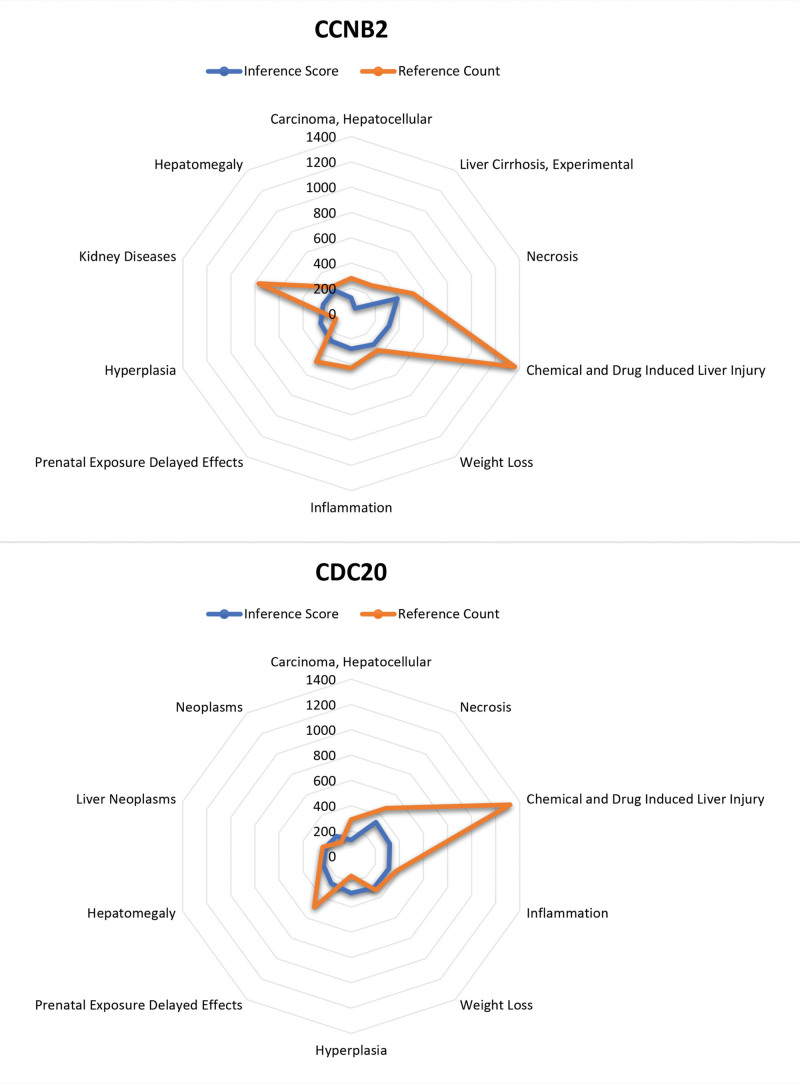
Comparative Toxicogenomics Database (CTD) analysis. (A) CCNB2. (B) CDC20.*P < .05, these genes were related to neoplasms, necrosis, inflammation, hyperplasia. CCNB2 = cyclin B2.

We entered list of hub genes into the CTD website to find diseases related to core genes, which improved understanding of gene-disease association. Two genes (CCNB2, CDC20) were found to be related to necrosis, inflammation, hyperplasia, and tumor (Fig. 12).

### 3.8. Prediction and functional annotation of miRNA related to core genes

We input list of hub genes into Targetscan to find relevant miRNA and improve understanding of gene expression regulation. We found that the related miRNA of the CCNB2 gene was hsa-miR-670-3p (Table 1).

**Table 1:** A summary of miRNAs that regulate hub genes.

	Gene	MIRNA
1	CCNB2	hsa-miR-670-3p
2	CDC20	None

CCNB2 = cyclin B2.

## 4. Discussion

The main result of this study was that CCNB2 was highly expressed in oral and bladder cancer, and the higher the CCNB2, the worse the prognosis.

CCNB2 is involved in G2/M transformation. The process involves the preparation of mitosis and the orderly allocation to the spindle.^[14]^ Uncontrolled mitosis can lead to tumor formation in various tissues, including oral and bladder cancers.^[15]^ Therefore, in recent years, research has found the important role of CCNB2 in tumors. For example, overexpression of CCNB2 is associated with poor prognosis in triple-negative breast cancer. The ability of invasion, migration, and proliferation of cancer cells decreased after knockdown expression.^[16]^ Wang^[17]^ confirmed that the upper and middle genes of lung adenocarcinoma can regulate the expression of CCNB2, thus inhibiting the progression of cancer.

In this study, high expression of CCNB2 was also found to regulate the progression of oral and bladder cancer. Further based on the sample data obtained by Gene Expression Omnibus, it was verified that CCNB2 is highly expressed in oral and bladder cancer, suggesting that high expression of CCNB2 is a cancer-promoting gene.^[18]^ CCNB2 is a key prognostic factor in patients with oral and bladder cancer. CCNB2 is a member of the cyclin family that regulates the cell cycle in eukaryotes by activating CDC2 kinase, and inhibition of CCNB2 induces cell cycle arrest.^[19]^ CCNB2 has been reported to be overexpressed in a variety of human cancers, such as endometrial cancer, skin cancer, prostate cancer, and gastric cancer. Abnormal expression of CCNB2 malregulates spindle checkpoints in the cell cycle and leads to chromosomal instability, one of the signature phenotypes of most cancers.^[20–22]^

CCNB2 regulates the activity of cyclin-dependent kinases. It is well known that different cyclins can regulate the eukaryotic cell cycle through CDK at specific points in the mitotic cycle.^[23]^ The progression of the cell cycle also follows cycles of alternating cyclin levels. Dysregulation of cyclin levels has been frequently observed in cancer. Elevated levels of cyclin E have been reported to be significantly associated with disease-specific survival in tumor patients.^[24,25]^ Amplification and/or overexpression of cyclin D1 is associated with poor prognosis in breast cancer patients. Upregulation of cyclin A can increase the risk of recurrence in tumor patients. According to previous reports, CCNB1 and CCNB2 play different roles in mitosis due to their significant differences in subcellular localization.^[26,27]^ In the interphase, CCNB2 is mainly associated with the Golgi apparatus, while CCNB 1 is colocalized with microtubules. In mitotic cells, CCNB 1 binds to chromosomes and is tightly bound to the spindle. CCNB2 is mainly distributed throughout the cell, and although a small fraction of CCNB2 is localized to the spindle, CCNB2 never binds to the chromosome.^[28,29]^ The localization of CCNB2 suggests that CCNB2 plays a role in regulating cell membrane transport during mitosis. When the cell enters mitosis, membrane transport is significantly inhibited and the Golgi apparatus disintegrates. CCNB2 often triggers the G2/M transition process by activating CDK 1.^[30,31]^ Downregulation of CCNB2 inhibits cell proliferation and promotes G2/M phase cell cycle arrest. Studies have shown that the dimethyl double muscle can downregulate the expression of CCNB2 to increase the rate of apoptosis and cell cycle arrest.^[32]^ High level of CCNB2 is positively correlated with the degree of undifferentiated tumor tissue, diameter, lymph node status, distant metastasis, and clinical stage. Therefore, we speculate that CCNB2 is highly expressed in oral and bladder cancer, promoting the proliferation of tumor cells, and thus worsening the disease.^[33]^

Although the value of CCNB2 in oral cancer and bladder cancer was analyzed with the help of the database, there are some limitations in the current study. The relevant sites of action of CCNB2 need to be further studied by human specimens and cell lines.

In summary, CCNB2 was one common oncogene of bladder cancer and OSCC. And CCNB2 was upregulated in oral cancer and bladder cancer, which might be one potential biomarker of the 2 cancers.

## Author contributions

**Conceptualization:** Lei Zhang.

**Formal analysis:** Bin Liu.

**Investigation:** Bin Liu.

**Methodology:** Bin Liu, Jianzhi Su.

**Project administration:** Bin Liu, Jianzhi Su.

**Resources:** Bin Liu.

**Software:** Lei Zhang.

**Supervision:** Jianzhi Su.

**Validation:** Jianzhi Su.

**Visualization:** Jianzhi Su.

**Writing – original draft:** Lei Zhang.

**Writing – review & editing:** Lei Zhang.
